# The first 25 years of the Northwestern University SuperAging Program

**DOI:** 10.1002/alz.70312

**Published:** 2025-08-07

**Authors:** Sandra Weintraub, Tamar Gefen, Changiz Geula, M‐Marsel Mesulam

**Affiliations:** ^1^ Mesulam Institute for Cognitive Neurology and Alzheimer's Disease Northwestern Feinberg School of Medicine Chicago Illinois USA; ^2^ Department of Psychiatry and Behavioral Sciences Northwestern Feinberg School of Medicine Chicago Illinois USA; ^3^ Department of Cell and Developmental Biology Northwestern Feinberg School of Medicine Chicago Illinois USA; ^4^ The Ken and Ruth Davee Department of Neurology Northwestern Feinberg School of Medicine Chicago Illinois USA

**Keywords:** aging, Alzheimer's disease, cognition, episodic memory, superaging

## Abstract

During late life, “average” does not mean “intact.” For example, cross‐sectional data from a common word list learning test show that average delayed word recall raw score at age 80 (5/15) is approximately half that at age 56 to 66 (9/15). Cognitive and neurobiological dissolution is therefore implicitly incorporated into concepts of the aging brain. This position is being challenged through investigations on “superaging,” a term that was coined at the Northwestern Alzheimer's Disease Research Center (ADRC) to define persons ≥ 80 years with delayed word recall raw scores *at least equal* to those of individuals 20 to 30 years younger. During the first 25 years of this program we established that superagers constitute not only a neuropsychological but also a neurobiological phenotype distinctive from cognitively average age peers. With respect to brain structure, superagers have cortical volumes no different than neurotypical adults 20 to 30 years younger in contrast to neurotypical peers who do show such age‐related shrinkage; they also have a region in the cingulate gyrus that is thicker than younger neurotypical adults. With respect to cellular biology, superagers have fewer Alzheimer's disease–type changes in the brain, greater size of entorhinal neurons, fewer inflammatory microglia in white matter, better preserved cholinergic innervation, and a greater density of evolutionarily progressive von Economo neurons. In the future, deeper characterization of the superaging phenotype may lead to interventions that enhance resistance and resilience to involutional changes considered part of average (i.e., “normal”) brain aging. This line of work is helping to revise common misperceptions about the cognitive potential of senescence and has inspired investigations throughout the United States and abroad.

**Highlights:**

- “Normal cognitive aging” is a term that spans a broad spectrum from average for age to well beyond.
- “Superaging” at the Northwestern University Alzheimer's Disease Research Center (ADRC) refers to a unique cognitive and biological phenotype.
- *Post mortem* findings support resilience and resistance to neuropathologic changes of aging.

## 1 THEORETICAL AND HISTORICAL PERSPECTIVE

The human brain can be conceptualized as a non‐equilibrium dynamic system in which constructive and involutional plasticity proceed on a background of net negative entropy. Early in development and well into adulthood, functionally constructive plasticity dominates. In time, however, biological mechanisms that are poorly understood start to undermine constructive plasticity throughout the body. For example, a skin wound takes twice as long to heal at the age of 40 than at the age of 20.^1^ The brain is no exception. To cite one example, hippocampal CA1 regeneration after CA2‐3 lesions is significantly faster in young adult versus aged rats.^2^ This regressive effect of time can be accelerated or decelerated by the interactive influence of genetic background, epigenetic phenomena, and stochastic events such as blood pressure, nutrition, exposure to infectious disease, inflammation, oxidative stress, exercise, head trauma, education, psychosocial background, and many others. While these considerations apply to all organs of the body, the brain is uniquely vulnerable because its principal neurons are postmitotic and therefore more exposed to time. In addition to transmitting signals across trillions of synapses, neurons of the cerebral cortex transport vast loads of organelles up and down axons at a speed of ≈ 50 cm a day. They are also gluttons for metabolic energy, releasing heat to the environment as the cost of information processing and entropy reduction. Given the enormity of this work and the resultant wear and tear, it is not surprising that the aging brain has been said to lose weight, volume, neuronal size, synapses, and transmitters, while also accumulating microinfarcts, white matter rarefaction, amyloid deposits, inflammatory reactions, and neurofibrillary degeneration. Despite these presumably inescapable consequences of a long life, there are aged individuals who do continue to acquire lots of new information, navigate complex situations, display exemplary judgment, and occasionally even show signs of enhanced creativity during advanced senescence.^3^

Considering this multiplicity of variables, it is not surprising that brain aging has received dramatically different interpretations. For Shakespeare, old age is “last scene of all, that ends this strange eventful history, is second childishness and mere oblivion, sans teeth, sans eyes, sans taste, sans everything.” Cicero had a different opinion: “Those … who allege that old age is devoid of useful activity … are like those who would say that the pilot does nothing in the sailing of his ship, because, while others are climbing the masts, or running about the gangways, or working at the pumps, he sits quietly in the stern and simply holds the tiller. He may not be doing what younger members of the crew are doing, but what he does is better and much more important. It is not by muscle, speed, or physical dexterity that great things are achieved, but by reflection, force of character, and judgment; in these qualities old age is usually not … poorer, but is even richer.”

Neurobiology has had little to say about Cicero's version, while Shakespeare's version has become dominant, paradoxically because of dramatic advances in understanding the biology of Alzheimer's disease (AD) and its linkage to aging. As a corollary to these advances, the prevailing views on brain aging focused on the potential mishaps of a long life and unwittingly muddied the distinction between neurotypical aging and AD. This state of affairs was fueled by epidemiologic research that showed a linear increase of AD dementia with age.^4^, ^5^, ^6^, ^7^, ^8^, ^9^ Furthermore, neuropathologic research revealed that the vast majority of cognitively neurotypical individuals beyond the age of 65 to 70 displayed at least some neurofibrillary tangles and amyloid beta (Aβ) plaques, the two histopathologic markers of AD.^10^, ^11^ When this information became added to the universally acknowledged age‐related (so‐called “benign”) decline of memory capacity, the fear arose that all aging would lead, sooner or later, to AD. The Northwestern University SuperAging Program was initiated in reaction to this prevailing mindset by addressing two principal questions. Is it possible to escape the purportedly universal age‐related erosion of brain capacity; and, if so, are such occurrences related to an identifiable biological phenotype?

The seeds of the Northwestern University SuperAging Program were sown by serendipity in the mid 1990s when we received the *post mortem* brain autopsy of an 81‐year‐old woman who had participated in a longitudinal research project led by Dr. Deborah Mash in Miami. The participant had shown no evidence of functional impairment and obtained memory scores that could be ranked as “superior” for her age, a level rated as psychometrically “average” for a 50‐year‐old. What was striking was the detection of only a single neurofibrillary tangle in a whole‐hemisphere section through the entorhinal cortex, a rare circumstance at that age, even for those with no known cognitive abnormality. The resultant implication that aging need not cause significant memory loss and that such a desirable outcome can be associated with a definable and distinctly non‐AD neuropathologic pattern led to the launching of the Northwestern University SuperAging Program with initial funding by The Davee Foundation in 2000 and 2001 and subsequently by the National Institute on Aging.

## 2 DEFINITION, RECRUITMENT, AND METRICS

We operationally defined superagers as persons who, at the age of ≥ 80, on the Rey Auditory Verbal learning Test (RAVLT), recalled at least 9/15 words after a delay, a score that was average for 56 to 66 years old, but considerably higher than the average for an 80+ year old (5/15).^12^ This is a high bar because the threshold we used is the average score expected from neurotypical persons *at least two to three decades younger* than superagers. The criteria also required scores on tests in all other cognitive areas to be at least at the average range for their age. Episodic memory function was chosen as the principal marker because it is the faculty with the most decline during average aging^13^, ^14^ and also the area of cognition that triggers frequent complaints in older individuals. The typographical space between “super” and “ager” was deliberately eliminated to indicate that the “superaging” term should be taken as a specific quantitative/operational designation, a symbolic trademark, rather than a generic descriptive phrase.

The initial recruitments came from the group of cognitively normal, “control,” participants enrolled in the Northwestern ADRC Clinical Core and also from word‐of‐mouth referrals (e.g., the grandmother of a colleague). The latter type of referral turned out to be overly optimistic, and dozens were considered “normal for age” but only ≈ 10% were considered “super for age” by our definition. Eventually, a steady stream of suitable participants was established through outreach activities for a total of 290 participants. At the time of this writing, the Northwestern ADRC Clinical Core contains 133 active participants. The mean age for those who entered as superagers (*N* = 101) is 90.1 (range 81–111 years.) Mean age for those who entered as neurotypical controls (*N* = 32) is 89 years (range 81–97 years.) The baseline mean RAVLT delayed score was 10.77 words (standard deviation [SD] = 2.51) for the superagers and 5.7 (SD = 1.96) for neurotypical peers. All other baseline cognitive test scores were at the age‐appropriate level in both participant groups. There have been 77 brain autopsies completed to date on participants via the Neuropathology Core of the Northwestern ADRC.

**FIGURE 1:**
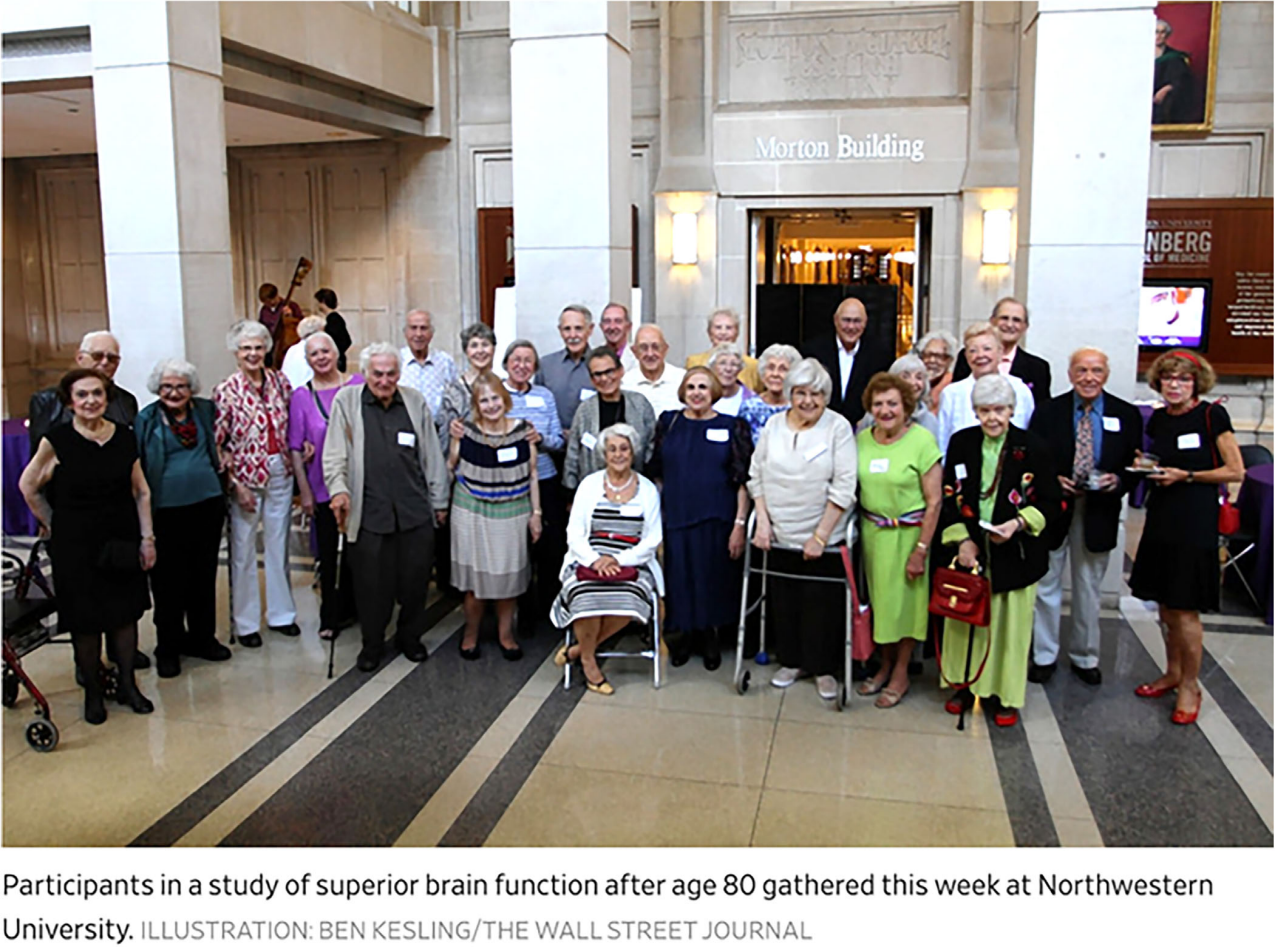
Participants in the Northwestern University SuperAging study gathered on May 24, 2013, to mingle and socialize. Photograph by Ben Kesling, at the time with *The Wall Street Journal*.

No particular lifestyle was conducive to superaging. Some superagers appeared to follow all conceivable recommendations for a healthy life. Others did not eat well, enjoyed smoking and drinking, shunned exercise, suffered stressful life situations, and did not sleep well. Superagers also did not seem to be medically healthier than their peers, as evinced by the similarity of medication regimens in neurotypical and superager groups.^15^ But the group was particularly sociable and relished extracurricular activities. Compared to their cognitively average, same‐aged peers, they rated their relationships with others more positively.^16^ Similarly, on a self‐reported questionnaire of personality traits they tended to endorse high levels of extraversion.^17^ Some Superagers demonstrated remarkable stability in cognitive performance over time; others declined but generally remained within the “average” range for age.^18^ The one observation that could be generalized was the gregariousness of the participants (see Figure 1).

## 3 SURPRISES FROM NEUROIMAGING

We found, as expected, that neurotypical seniors ≥ 80 years of age have significant and widespread cortical thinning compared to neurotypical 50‐ to 60‐year‐olds.^19^ Superagers, on the other hand, showed no cortical thinning compared to the younger controls. In other words, belonging to a group of individuals who obtained a score of ≥ 9 on the RAVLT at the age of ≥ 80 appears, by itself, to indicate membership in an exclusive group in which cerebral cortex does not conform to age‐related thinning norms.

Does this result mean that superagers start life with larger brains? Because it is not possible to have retroactive brain imaging, the indirect way to address this question is through longitudinal studies. In a preliminary study over 18 months, overall cortical thickness was reduced by 1.06% in superagers compared to 2.24% in neurotypical peers, a difference that was statistically significant.^20^ It appears that cortical thinning is unavoidable, but that it is probably much slower in superagers. Whether these individuals are also born with larger brains remains to be addressed, but is unlikely to be the entire answer given the absence of obvious differences in skull morphology.

**FIGURE 2:**
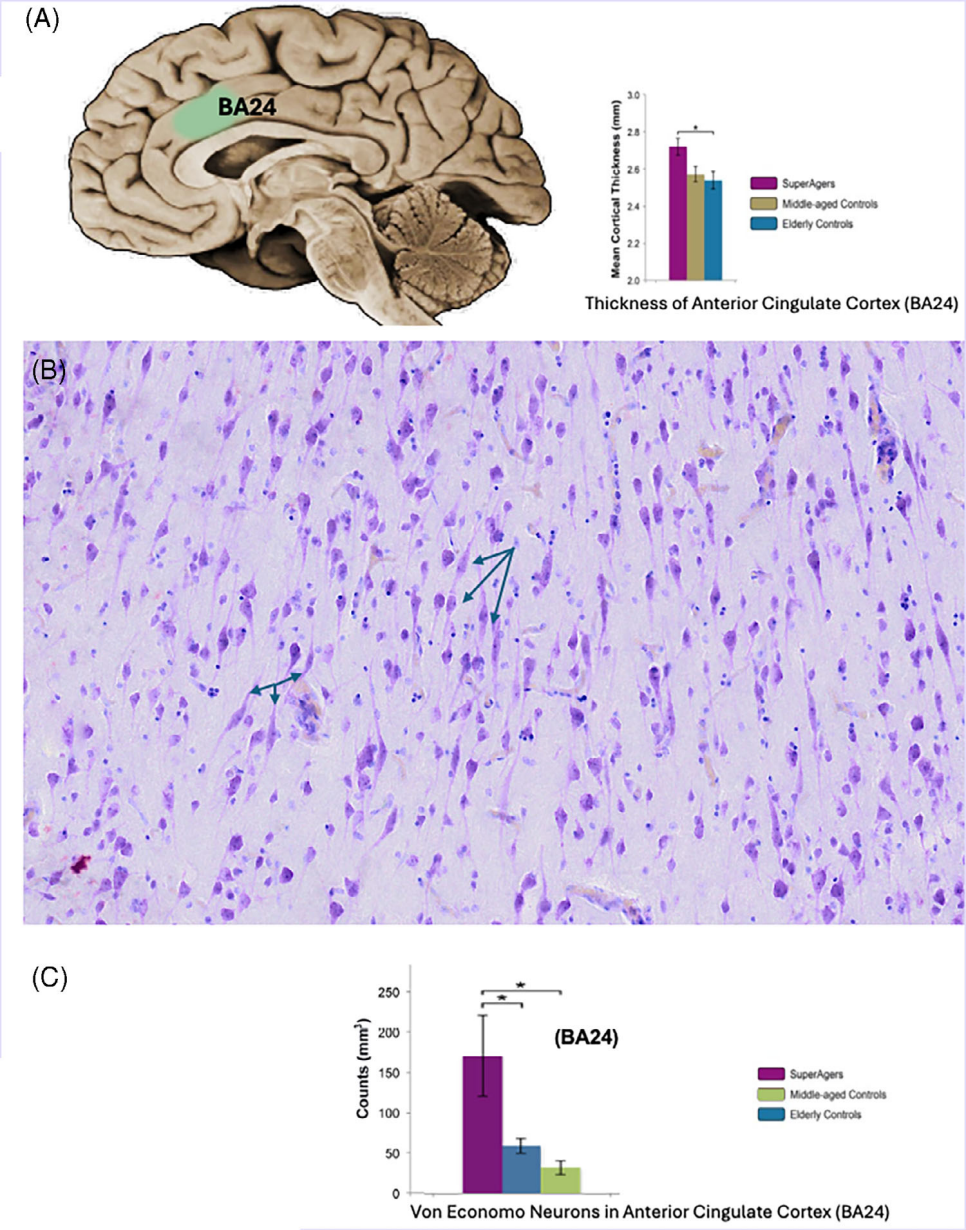
The anterior cingulate cortex displays selective properties in superagers. (A) The green area in the rendering of the brain on the left depicts the anterior cingulate cortex (Brodmann area 24 [BA24]), which in quantitative magnetic resonance imaging studies was found to be thicker in superagers not only compared to age‐matched neurotypical controls but also compared to a cohort of normal individuals 20 to 30 years younger. Bar graph on the right shows the quantitative analysis of cortical thickness in the three groups. (B) Micrograph of Nissl‐stained neurons in the anterior cingulate cortex of a superager brain, visualizing a population of von Economo neurons (arrows). Von Economo neurons are found in considerably higher density in superagers. (C) Quantitative stereological counts confirm significantly higher density of von Economo neurons in superagers compared to neurotypical elderly or middle‐aged individuals. The anterior cingulate cortex displays selective properties in superagers.

The most surprising finding was the identification of an anterior cingulate region in which superagers had greater cortical thickness than even neurotypical participants 50 to 60 years of age (Figure 2A). This finding subsequently has been confirmed in other studies.^21^, ^22^, ^23^, ^24^ The anterior cingulate is a primary component of the salience and anterior paralimbic networks, which mediate processes related to homeostasis; motivation; emotion; and, most importantly, social networking and affiliative behaviors, factors that resonate with superager characteristics.^25^, ^26^

## 4 SURPRISES FROM NEUROPATHOLOGY

### 4.1 Von Economo neurons in the anterior cingulate gyrus

The cingulate gyrus is one of the few regions in the brain that contains aggregates of nerve cells known as von Economo neurons. As shown in Figure 2B from the *post mortem* brain of a superager, von Economo neurons are distinctive spindle‐shaped nerve cells. They are most conspicuous in the anterior cingulate and frontoinsular regions of brains from cognitively normal individuals.^27^, ^28^, ^29^, ^30^ In addition to being thicker, the anterior cingulate gyrus of superagers also contains more von Economo neurons compared not only to neurotypical peers but also to much younger persons (Figure 2B,C).^29^, ^30^, ^31^ Phylogenetically, the most prominent collections of von Economo neurons occur in the brains of humans, great apes, cetaceans, and elephants, species with extensive affiliative behaviors,^27^, ^28^ which, as noted above, also characterize superagers. The density of von Economo neurons did not show age‐related changes in neurotypical seniors, suggesting that superagers may be born with a higher density of these neurons.

### 4.2 Neurofibrillary degeneration

**FIGURE 3:**
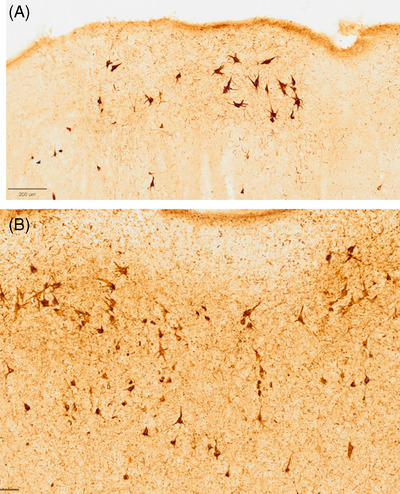
(A) Superagers display significantly lower density of neurofibrillary tangles and pre‐tangles in the entorhinal cortex compared to neurotypical elderly (B).

The research of the 1980s and 1990s was heavily focused on AD. It soon became clear that deposits of hyperphosphorylated tau rather than amyloid plaques were most tightly correlated with cognitive impairment.^32^, ^33^ It was also shown that scattered tau‐positive neurofibrillary tangles in^34^ entorhinal–hippocampal cortices were ubiquitous in average aging, raising the possibility that they might be responsible for the neurotypical age‐related weakening of memory.^10^ Initial questions were therefore related to the state of AD‐like neurofibrillary changes in superagers (Figure 3). We found that neurofibrillary tangle density at the lowest Braak stages (0–I) was found only in superagers; that only peers with average age‐related memory decline had neurofibrillary degeneration of moderately high intensity (stage IV), and that stages of moderate intensity (Braak II–III) occurred in both superagers and neurotypical peers.^35^ Stereological quantitative methods confirmed that, as a group, superagers have significantly fewer neurofibrillary tangles in rhinal cortices than cognitively average peers. These findings led to the conclusion that there are at least two pathways to the maintenance of youthful memory capacity in old brains, namely *resistance* to the emergence of neurofibrillary pathology and *resilience* to the cognitive impact of neurofibrillary pathology. Along this line of research, we found that the size of entorhinal layer two neurons (which provide the major source of input into the hippocampus through the perforant pathway) is larger in superagers.^36^ This feature may make the entorhinal–hippocampal pathway constitutively more resistant to involutional changes, including neurofibrillary degeneration. Alternatively, this difference in size may indicate reactive and constructive cellular plasticity that leads to resilience in the face of pathology. Furthermore, preliminary analysis of plasma biomarkers revealed lower levels of tau phosphorylated at residue 181 (p‐tau181) in superagers, consistent with reduced neurofibrillary degeneration in these individuals.^37^

**FIGURE 4:**
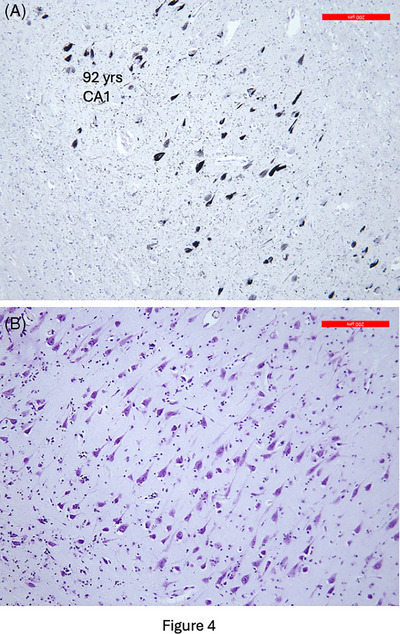
(A) Bielschowsky silver stain visualized a moderate density of tangles in the CA1 sector of the hippocampus in a 95‐year‐old superager. (B) Despite the presence of tangles, Nissl stain visualized an abundance of healthy appearing neurons in this region.

The investigations of the superaging brain have also raised conceptual questions related to the interpretation of Braak staging. This ranking system of neurofibrillary degeneration dominates the neuropathologic characterization of AD and its distinction from average aging. The staging is based on the density of neurofibrillary tangles rather than of residual viable neurons. Figure 4 shows prominent neurofibrillary tangles in the hippocampal CA1 sector of a 92‐year‐old superager. The cresyl violet stain of the same area shows a much greater population of normal‐looking neurons. To rank this specimen at Braak stage II overlooks the spared neurons, which may well be playing the decisive role in memory function compared to neurotypical peers who may also be at Braak stage II but with fewer viable neurons.

### 4.3 Basal forebrain cholinergic system

The computations of the cerebral cortex revolve around two types of connections, *channel* connections that link a specific cortical region to another cortical or subcortical region, and *state* connections that originate in small groups of subcortical neurons and innervate the entire cerebral cortex. The latter group plays a key role in modulatory functions related to attention, signal‐to‐noise ratio, mood, emotion, motivation, and so forth. Neurons in the basal forebrain provide the most substantial of state connections and invest all parts of the cerebral cortex with a dense network of cholinergic axons.^38^, ^39^, ^40^, ^41^, ^42^ Cholinergic pathways are thought to have important modulatory influences on attention and memory. For example, the cholinergic inhibitor scopolamine is known to impair memory. The basal forebrain cholinergic neurons are also among the first to undergo neurofibrillary degeneration in otherwise neurotypical aging and may partially underlie the weakening of memory in average aging.^43^ The number of these neurons is significantly reduced in AD and causes a depletion of cholinergic projections to the cerebral cortex.^34^, ^44^ It is therefore quite interesting that basal forebrain cholinergic neurons showed significantly fewer neurofibrillary tangles in superagers than in neurotypical peers. Furthermore, axonal abnormalities, such as ballooning or thickening, also appeared less abundant in superagers than neurotypical controls.^45^

In the cerebral cortex, acetylcholine released by presynaptic varicosities along cholinergic axons contact cholinoceptive cortical neurons through muscarinic and nicotinic receptors. A subset of these cholinoceptive neurons stand out because of an unusually high content of the hydrolytic enzyme acetylcholinesterase.^43^ This enzyme terminates the synaptic action of acetylcholine through the very rapid hydrolysis of the transmitter. In fact, the drugs most frequently used for AD exert their therapeutic effects by inhibiting acetylcholinesterase to enhance the postsynaptic impact of acetylcholine. Surprisingly, the density of these acetylcholinesterase‐rich neurons was significantly lower in superagers compared to neurotypical controls.^46^ This finding could be associated with reduced degradation of acetylcholine in superagers, potentially increasing the postsynaptic impact of this neurotransmitter. It appears, therefore, that the functionality of the cortical cholinergic system is enhanced in superagers at the neuronal, axonal, and synaptic levels. Whether this is inherited or acquired through reactive plasticity remains to be determined. One prediction that comes out of these findings is that superagers should be more resistant to the effects of scopolamine on memory.

### 4.4 Microglia

**FIGURE 5:**
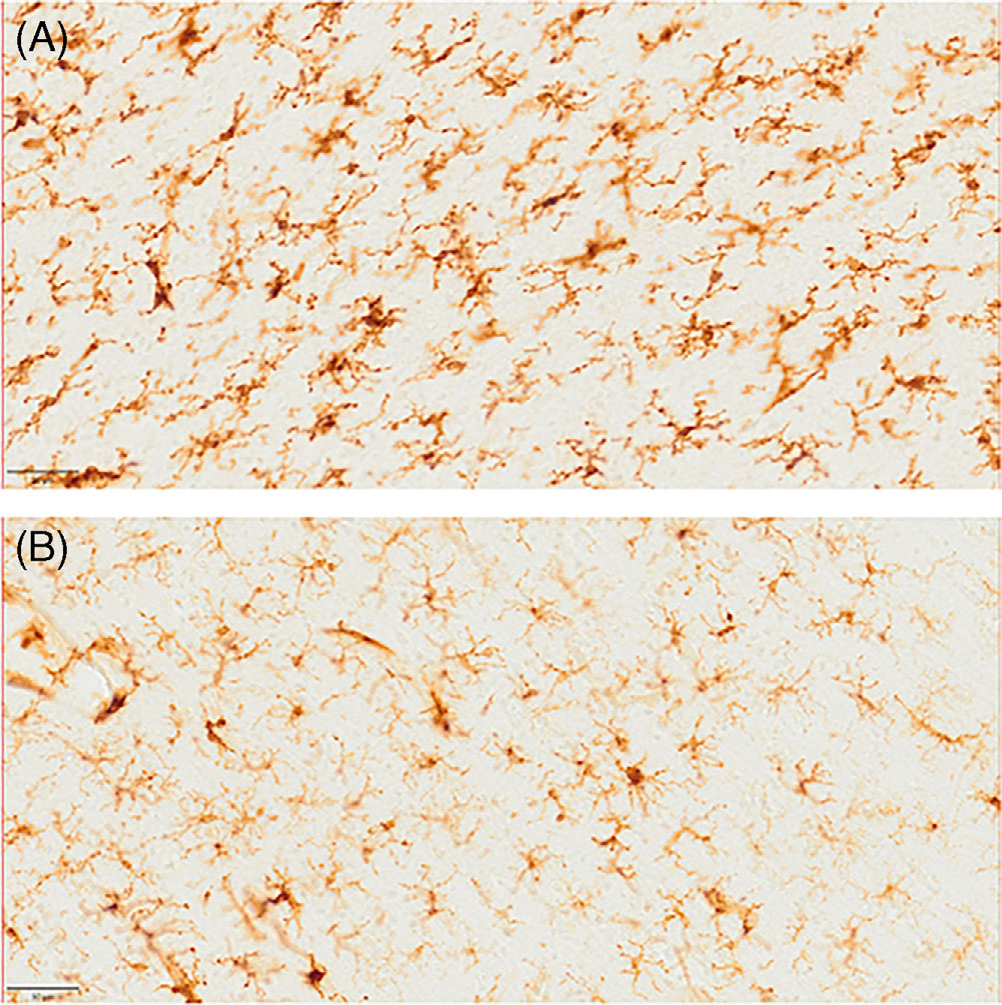
Immunostaining for HLA‐DR, a specific marker of activated microglia, visualizes a high density of these cells in frontal white matter in neurotypical elderly (A). Superager white matter (B) contains significantly fewer activated microgliaImmunostaining for HLA‐DR, a specific marker of activated microglia, visualizes a high density of these cells in frontal white matter in neurotypical elderly.

Microglia can be helpful or harmful. They play an important role in promoting or undermining plasticity by participating in synaptic pruning and stimulating inflammatory processes. Activation of microglia in white matter is part of physiological aging.^47^ Furthermore, nearly all known neurodegenerative diseases causing dementia are associated with intense microgliosis. It is therefore quite interesting that activated microglia are less numerous in the white matter of superagers than in neurotypical brains (Figure 5). Furthermore, preliminary findings indicate that microglia isolated from superager cortex display unique characteristics and different rates of proliferation in culture when isolated from *post mortem* brains.^48^, ^49^ The impact of this phenomenon on cortical involution remains to be determined.

## 5 ILLUSTRATIVE CASE STUDY OF A NORTHWESTERN SUPERAGER FOLLOWED FOR 25 YEARS

A right‐handed White woman enrolled into longitudinal research at the Northwestern ADRC as a healthy neurotypical participant in her 60s. At the age of 80, she met criteria for being labelled a superager. She had a master's degree in social work. Medical history included basal cell carcinoma, hip replacement, and hysterectomy. She was diagnosed with late stage cancer and received chemotherapy, but metastases were identified 6 months later. She subsequently had an ischemic stroke and was discharged to hospice where she died at the age of 84.

Up to the time of the stroke she was entirely independent in daily living and remained highly engaged in her community. There was no history of significant alcohol, tobacco, or other substance use. Family history was significant for Parkinson's disease in a parent who died at age 90. Delayed recall score on the RAVLT was 11/15 at the age of 67 years and also 11/15 at the age of 82 years, 15 years later, at an age when the neurotypical cutoff for normalcy is 5/15. At the age of 68, she correctly named 93% of objects she was shown, and 88% at the age of 82, 14 years later. Her reverse digit span was 6 at the age of 70 and 7 at the age of 83, 13 years later.

**FIGURE 6:**
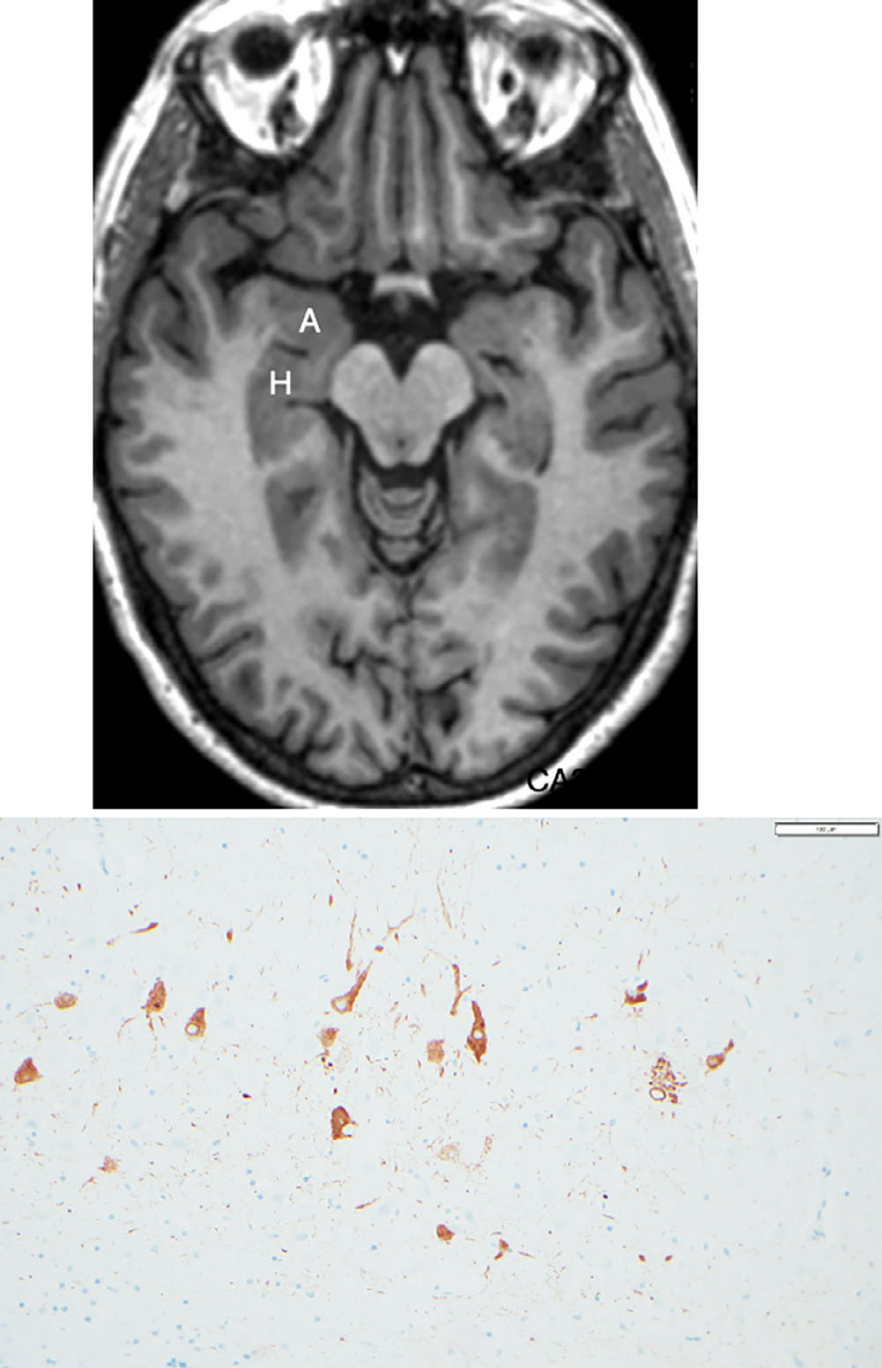
Magnetic resonance imaging scan of a superager (top) did not show significant atrophy or ischemic changes. In specific, the amygdala (A) and hippocampus (H) were entirely normal even for a young person. Phosphorylated tau immunostaining (bottom) showed relatively low density of neurofibrillary tangles and pretangles, consistent with Braak stage II of neurofibrillary degeneration. Bottom figure courtesy of Rudy Castellani, MD.

In keeping with this cognitive stability and unusually high performance, serial structural magnetic resonance imaging collected during research visits did not show significant atrophy or ischemic changes. In particular, the amygdala and hippocampus were entirely normal even for a young person (Figure 6A). Her apolipoprotein E (*APOE*) status was ε3/ε3. On *post mortem* examination, there was no evidence of gross atrophy. p‐tau immunostains of the entorhinal cortex and hippocampus showed scattered and sparse pretangles in the entorhinal cortex and the CA2 subfield of the hippocampus, consistent with Braak stage II (Figure 6B). The rest of the cerebral cortex was virtually free of p‐tau pathology. There was no appreciable Aβ pathology. There was no synucleinopathy, TAR DNA‐binding protein 43 proteinopathy, or tau astrogliopathy, all of which are commonly seen in the senescent brain of cognitively normal aged individuals.  Inspection of the anterior cingulate gyrus revealed many von Economo neurons.

## 6 CONCLUSIONS

Over the first 25 years of the Northwestern University SuperAging Program, we showed that it is possible to identify groups of persons who clinically seem to have avoided the memory decline of average aging and who display biological markers that separate them from average agers. These superagers maintain good brain morphology, tend to be gregarious, appear resistant to neurofibrillary degeneration and resilient to its consequences, have a more robust cholinergic system, carry more von Economo neurons, and have less inflammatory microglial activity in the white matter. These features are starting to coalesce into a clinicobiological phenotype that can be investigated in depth, with significant implications for clarifying, by contrast, the regressive pressures upon brain and memory during what is otherwise deemed normal aging.

As mentioned above, the brain is a site of both constructive neuroplasticity and erosive phenomena. The constructive forces are ascendant early in life but gradually lose dominance, the slope of the reversal being determined by genetic, epigenetic, and environmental factors. AD is an extreme example of involutional dominance. Even average cognitive aging, however, is associated with a diminution of brain substance and a resultant weakening of memory capacity, but not to the extent of interfering with most age‐appropriate functions. The superaging phenotype appears to represent a state in which progressive factors promoting plasticity enable relative resistance and resilience to at least some of the factors that mediate the involutional impact of aging. Naturally, all good things do come to an end and involutional processes in the brain eventually take over if longevity permits. One obvious question is whether the regressive correlates of average aging can be slowed and delayed to promote the superaging phenotype. Preliminary evidence suggests that genes such as *KLOTHO, BDNF, APOE, REST*, and *TMEM106b* might exert such influences.^50^, ^51^, ^52^ Their relevance to superaging remains to be investigated.

Although many questions remain to be addressed, the findings so far establish the remarkable fact that remembering 9 words out of a list of 15 at age 80 serves as a marker of a distinct neurobiological signature related to brain structure and function. Even if it turns out that the principal factor promoting superaging is inborn, the identification of associated genetic factors could potentially lead to pharmaceutically modifiable protein targets that promote the longevity of cognition and its protection from AD.

## CONFLICT OF INTEREST STATEMENT

Sandra Weintraub has no conflicts to report. Tamar Gefen has no conflicts to report. Changiz Geula has no conflicts to report. M‐Marsel Mesulam has no conflicts to report. Author disclosures are available in the supporting information.

## CONSENT STATEMENT

Consent was not necessary as this paper reports on completed research reported in other publications that contain statements of consent as appropriate for each paper.

## Supporting information



Supporting information
